# Comparative cost-effectiveness analysis of CDK4/6 inhibitors in the first-line treatment of HR-positive and HER2-negative advanced breast cancer: a Markov's model-based evaluation

**DOI:** 10.3389/fonc.2024.1413676

**Published:** 2024-07-24

**Authors:** Shereen Elazzazy, Nour Hisham Al-Ziftawi, Mohamed Izham Mohamed Ibrahim, Salha Bujassoum, Anas Hamad

**Affiliations:** ^1^ Pharmacy Department, National Center for Cancer Care & Research, Hamad Medical Corporation, Doha, Qatar; ^2^ Pharmacy Department, Aman Hospital, Doha, Qatar; ^3^ College of Pharmacy, QU Health, Qatar University, Doha, Qatar; ^4^ Oncology Department, National Center for Cancer Care & Research, Hamad Medical Corporation, Doha, Qatar

**Keywords:** breast cancer, palbociclib, ribociclib, abemaciclib, cost-effectiveness

## Abstract

**Introduction:**

CDK4/6 inhibitors are the first-line treatment for HR+/HER2- advanced breast cancer. Despite their clinical benefit, they can increase healthcare expenditure. To date, there is no thorough comparison among the three approved CDK4/6 inhibitors in terms of their cost-effectiveness.

**Objective:**

To investigate and compare the cost-effectiveness of CDK4/6 inhibitors in combination with letrozole as a first-line treatment for advanced breast cancer with hormonal-receptor-positivity and HER-2-negativity versus one another and versus letrozole monotherapy.

**Methods:**

A 10-year within-cycle-corrected Markov’s model was employed from the healthcare payer perspective. Costs were obtained from the National Center for Cancer Care and Research (NCCCR) in Qatar. Utilities and transition probabilities were calculated from published landmark trials of PALOMA-2, MONALEESA-2, MONARCH-3, PO25, and other relevant literature. Costs, measured in Qatari Riyal (QAR), and effectiveness, measured in quality-adjusted-life-years (QALYs), were incremented and the incremental cost-effectiveness ratio (ICER) was compared to a willingness-to-pay threshold (WTP) of 1.5 Qatari GDP (448,758 QAR). A deterministic sensitivity analysis was implemented to account for uncertainties.

**Results:**

Ribociclib was the most effective option, generating 4.420 QALYs, followed by palbociclib (4.406 QALYs), abemaciclib (4.220 QALYs), then letrozole monotherapy (2.093 QALYs). As for cost-effectiveness, ribociclib dominated palbociclib. However, it was not cost-effective compared to abemaciclib (ICER=1,588,545 QAR/QALY). Ribociclib remained dominant over palbociclib with all uncertainties. The base-case conclusion of ribociclib versus abemaciclib remained robust over all uncertainties.

**Conclusion:**

From the healthcare payer perspective in Qatar, ribociclib is the most effective CDK4/6 inhibitor. It was dominant over palbociclib in terms of cost-effectiveness; however, it was not cost-effective compared to abemaciclib at current prices.

## Highlights

- Healthcare resources are scarce and therefore, their use should be guided using health economics-based evidence.
- CDK4/6 inhibitors are nowadays the mainstay for HR+/HER2- advanced breast cancer.
- Most cost-effectiveness evaluations focus on CDK4/6 inhibitors vs. endocrine monotherapy, or palbociclib vs. ribociclib only.
- This study can guide clinicians and decision-makers in similar economies about the best use of CDK4/6 inhibitors in first-line treatment of HR+/HER2- advanced breast cancer.
- Further research is granted into the economic impact of treatment sequencing and identifying patient subgroups that drive maximal benefit from specific CDK4/6 inhibitors.

## 1 Introduction

Cyclin-dependent kinase 4/6 (CDK4/6) inhibitors are the first-line treatment for hormone receptor-positive (HR+), human epidermal growth factor receptor 2-negative (HER2-) advanced breast cancer in the absence of visceral crisis (1). There are three CDK4/6 inhibitors that are currently approved by the Food and Drug Administration (FDA) for the treatment of advanced breast cancer: palbociclib, ribociclib, and abemaciclib (2). All three drugs are highly effective in improving progression-free survival (PFS) for patients with advanced HR+/HER2- breast cancer in combination with their indicated FDA combinations tamoxifen, fulvestrant, or aromatase Inhibitors (AIs) such as anastrozole and Letrozole (3–9). However, in terms of the overall survival (OS), it is only ribociclib and abemaciclib that were clinically proven to prolong OS in HR+/HER2- advanced breast cancer patients (6, 10–12). Although CDK4/6 inhibitors were proven clinically effective, they can increase healthcare expenditure due to the high drug acquisition cost and due to the cost of monitoring or supportive care associated with adverse drug reactions (13). Since resources are scarce, it is important to utilize them wisely to ensure sustainability of functioning (14). This includes healthcare resources too. In fact, drug therapeutic options and their costs are estimated to range from 9% to 20% of the total health expenditures in many countries (15). Therefore, the use of such expensive medications as CDK4/6 inhibitors needs to be evidence-based and supported by cost-effectiveness evidence.

To date, the three CDK4/6 inhibitors plus endocrine were not found to be cost-effective in comparison to endocrine monotherapy in several settings (16). However, since they are now recommended to be the mainstay therapy for HR+/HER2- advanced breast cancer, their cost-effectiveness should be compared to each other if they are to be included in the formulary. In comparison to each other, ribociclib was found to be more cost-effective than palbociclib in different settings from the United States (US), Spain, and Qatar (13, 17–19). With regards to abemaciclib, to our knowledge, there are only two studies that addressed its cost-effectiveness compared to other CDK4/6 inhibitors in advanced breast cancer, and both were carried out in the US. For the first one, the use of abemaciclib plus fulvestrant was not cost-effective compared to palbociclib plus fulvestrant (20). However, for the second one, abemaciclib was found to be cost-effective compared to ribociclib in the second-line treatment for HR+/HER2- advanced breast cancer in the US (21). However, to our knowledge, there are no yet thorough comparative cost-effectiveness evaluations comparing the three of the CDK4/6 inhibitors together in the same settings.

To date, there is a general lack and need for robust pharmacoeconomic evaluations of breast cancer medications in developing countries (22). Qatar is an independent Gulf country that is classified as having a developing economy according to the United Nations classification (23). In Qatar, there is only one cost-effectiveness study that was carried out regarding the use of CDK4/6 inhibitors in HR+/HER2- advanced breast cancer patients. However, this study was associated with some limitations in that it only addressed palbociclib and ribociclib without abemaciclib. In addition, it was based on observational real-world evidence, which can come with potential uncertainties due to the small sample size and short follow-up duration. Moreover, to our knowledge, there is no study comparing the cost-effectiveness of the three CDK4/6 inhibitors altogether in the first-line treatment of HR+/HER2- advanced breast cancer; the previously mentioned one was a comparison in the second-line treatment (21). Therefore, to fill these gaps in the literature, this study aims to provide comprehensive cost-effectiveness analysis for the three FDA-approved CDK4/6 inhibitors —palbociclib, ribociclib, and abemaciclib in combination with letrozole (being the most commonly used combination with CDK4/6 inhibitors in Qatar (24)) in the first-line treatment for HR+/HER2- advanced breast cancer women.

## 2 Materials and methods

### 2.1 Model overview

A Markov model was constructed to evaluate the cost-effectiveness of CDK4/6 inhibitors in combination with letrozole in the first-line treatment of HR+/HER2- advanced breast cancer to each other and to letrozole monotherapy. The model consisted of three health states: progression-free (PFS), progressed disease (PD), and death. Patients were initially placed in the PFS health state and could transition to the PD health state at any time, or to the death health state at any time from either the PFS or PD health states. The model was simulated over a 10-year time horizon. The cycle length was one month, which is the recommended monitoring frequency during the treatment duration according to the drug monographs and guidelines. The model was also constructed with the following assumptions:

- patients would receive their CDK4/6 inhibitor or their letrozole monotherapy as a first-line treatment
- patients would enter the model with the full dose regimen for each of the treatment arms as follows: palbociclib 125 mg once a day for 21 days + Letrozole 2.5 mg once a day for 28 days for the palbociclib treatment arm, ribociclib 600 mg once a day for 21 days + Letrozole 2.5 mg once a day for 28 days for the ribociclib arm, abemaciclib 150 mg twice a day for 28 days + letrozole 2.5 mg once a day for 28 day, or Letrozole 2.5 mg once a day for 28 days.
- patients who develop grade 3/4 hematological or gastroenterological side effects, or QTC interval (>500 mm/sec), or grade 3 hepatotoxicity would undergo a mandatory dose reduction with one level as follows: palbociclib 100 mg once a day for 21 days + Letrozole 2.5 mg once a day for 28 days for the palbociclib arm, ribociclib 400 mg once a day for 21 days+ Letrozole 2.5 mg once a day for 28 days for the ribociclib arm, or abemaciclib 100 mg twice a day for 28 days + letrozole 2.5 mg once a day for 28 days. No dose reductions would be allowed in the letrozole group.
- for the next cycles, if the side effects mentioned in the previous point re-occur, a patient would undergo another reduction. No dose reductions would be allowed in the letrozole group.
- patients would discontinue the treatment and move to the next treatment line if any of the following happened: 1) the side effects mentioned in the previous two points persisted despite two levels of dose reduction, a patient developed grade 3 or 4 of QTC interval prolongation, or a patient developed grade 4 hepatotoxicity. 2) Progression of the disease.
- at the PD health state, patients would be placed on second-line hormone and/or chemotherapy according to the standard of care at the facility
- all patients would eventually die
- the transition between the three health states is unidirectional where a patient can move from PFS to PD or death, or from PD to death only.

The analysis was done from the payer perspective which is the governmental cancer-specialized hospital, The National Center for Cancer Care and Research (NCCCR), which is a part of the main governmental healthcare provider, Hamad Medical Corporation (HMC). The primary outcome of the model was the incremental cost-effectiveness ratio (ICER), which is the incremental cost per additional quality-adjusted life year (QALY) gained. The ICER was calculated by comparing the costs and QALYs of each of the three CDK4/6 inhibitors plus letrozole to letrozole alone as a first step. Then the model was re-run to compare the ICERs among the three different CDK4/6 inhibitors plus letrozole in comparison to each other. The model was carried out using TreeAge Pro (healthcare version) 2022 R2.1. All the ICERs were calculated in the units of Qatari Riyal (QAR) per QALY gained, where 1 US dollar is equal to 3.65 QAR according to the 2023 financial year. All ICERs were compared to a WTP threshold of 448,785 QAR, corresponding to 1.5 gross domestic product (GDP) per capita according to the International Monetary Fund 2023 (25).

### 2.2 Model Inputs

#### 2.2.1 Costs

The costs included in this model were based only on direct medical costs obtained directly from the HMC formulary for the medications, and from the HMC Department of Accounting and Finance for the related costs. All costs were based on the 2022–2023 financial year and were put into the model in the local currency of QAR. The first component of the direct medical costs was based on the drug acquisition cost for each of the treatment arms generated by the unit dose cost multiplied by the number of days consumed for each of the medication components. The other components of direct medical costs per cycle of treatment included the needed laboratory tests throughout the treatment period such as blood count (CBC), blood metabolic panel, liver function test, endocrinology lab tests, tumor markers and catechol amines, and coagulation tests. Moreover, it included the radiology costs such as any X-ray, ultrasound, mammogram, magnetic resonance imaging (MRI), computed tomography (CT), and positron emission tomography scan (PET scan) required during the treatment period in addition to the required cardiac monitoring required such as electrocardiogram (ECG) and echocardiogram. The hospitalization costs were also estimated and included in the cost calculations of the model. While the drug acquisition cost plays the essential pillar of the cost calculation per cycle, the cycle cost was deemed to be the summation of all the direct medical costs (per average number of units needed during the one-month cycle) obtained from the hospital payer perspective. For palbociclib, ribociclib, abemaciclib, and letrozole, the number of units needed of medications, laboratory tests, imaging, and hospitalization were obtained and averaged from the landmark trials, PALOMA, MONALEESA, MONARCH, and PO25 respectively. The costs used for this model input are summarized in **Table 1**.

**Table 1:** Inputs of Markov’s Model.

Input	Value	Source of Data	Data Source Reference
Cost Per Cycle (QAR)			
**PFS (Palbociclib 125 mg)**	20,215.75	RWD- HMC	–
**PFS (Palbociclib 100 mg)**	18,503.83	RWD- HMC	–
**PFS (Palbociclib 75 mg)**	20,215.75	RWD- HMC	–
**PFS (Ribociclib 600 mg)**	19,068.16	RWD- HMC	–
**PFS (Ribociclib 400 mg)**	13,578.97	RWD- HMC	–
**PFS (Ribociclib 200 mg)**	8,089.78	RWD- HMC	–
**PFS (Abemaciclib 150 mg)**	13,478.67	RWD- HMC	–
**PFS (Abemaciclib 100 mg)**	13,478.67	RWD- HMC	–
**PFS (Abemaciclib 50 mg)**	13,478.67	RWD- HMC	–
**PFS (Letrozole 2.5 mg)**	2,082.25	RWD- HMC	–
**PD**	3,531.52	RWD- HMC	–
Utility Values			
**PFS (Palbociclib- all doses)**	0.7507	PALOMA-2	(26)
**PFS (Ribociclib- all doses)**	0.774	MONALEESA-2	(27)
**PFS (Abemaciclib- all doses)**	0.745	MONARCH-3	(28)
**PFS (Letrozole)**	0.73	Report estimating utility value for advanced BC on hormonal therapy	(29)
**Grade 3/4 hematological toxicities and neutropenia**	0.72	A HR-QoL study evaluating neutropenia in cancer patients	(30)
**Grade 3/4 Diarrhea and GI SEs**	0.70	A study about HR-QoL in different subgroups of metastatic breast cancer	(31)
**Hepatotoxicity**	0.77	Study evaluating HR-QOL in patients with liver disease	(32)
**PD**	0.505	A study about HR-QoL in different subgroups of metastatic breast cancer	(31)
Transition Probabilities			
**PFS to PD (Palbociclib)**	0.0211	PALOMA-2	(3)
**PFS to death (Palbociclib)**	0.000779	PALOMA-2	(3)
**PD to death (Palbociclib)**	0.0067	PALOMA-2	(3)
**PFS to PD (Ribociclib)**	0.0195	MONALEESA-2	(5)
**PFS to death (Ribociclib)**	0.00031	MONALEESA-2	(5)
**PD to death (Ribociclib)**	0.0091	MONALEESA-2	(5)
**PFS to PD (Abemaciclib)**	0.0222	MONARCH-3	(8)
**PFS to death (Abemaciclib)**	0.00191	MONARCH-3	(8)
**PD to death (Abemaciclib)**	0.0058	MONARCH-3	(8)
**PFS to PD (Letrozole)**	0.0302	PO25 Trial	(33)
**PFS to death (Letrozole)**	0.0049	PO25 Trial	(33)
**PD to death (Letrozole)**	0.056	PO25 Trial	(33)
**Dose reduction by 1 level (palbociclib)**	0.0107	PALOMA-2	(3)
**Dose reduction by 2 levels (palbocilib)**	0.0066	PALOMA-2	(3)
**Drug Discontinuation (palbociclib)**	0.0044	PALOMA-2	(3)
**Dose reduction by 1 level (Ribociclib)**	0.027	MONALEESA-2	(5)
**Dose reduction by 2 levels (Ribociclib)**	0.014	MONALEESA-2	(5)
**Drug Discontinuation (Ribociclib)**	0.0051	MONALEESA-2	(5)
**Dose reduction of 1 level (Abemaciclib)**	0.0183	MONARCH-3	(34)
**Dose reduction by 2 levels (Abemaciclib)**	0.0092	MONARCH-3	(34)
**Drug Discontinuation (Abemaciclib)**	0.0121	MONARCH-3	(34)
**Dose reduction of 1 level (Letrozole)**	0	MONALEESA-2	(5)
**Dose reduction by 2 levels (Letrozole)**	0	MONALEESA-2	(5)
**Drug Discontinuation (Letrozole)**	0	MONALEESA-2	(5)
**Grade 3/4 Neutropenia (Palbociclib)**	0.04636	PALOMA-2	(3)
**Grade 3/4 Neutropenia (Ribociclib)**	0.05934	MONALEESA-2	(5)
**Grade 3/4 Neutropenia (Abemaciclib)**	0.01323	Safety report based on MONARCH-2 and MONARCH-3	(35)
**Grade 3/4 Neutropenia (Letrozole)**	0.00059	MONALEESA-2	(5)
**Grade 3/4 Diarrhea (Palbocilcib)**	0.0009	PALOMA-2	(3)
**Grade 3/4 Diarrhea (Ribociclib)**	0.002	MONALEESA-2	(5)
**Grade 3/4 Diarrhea (Abemaciclib)**	0.0122	Safety report based on MONARCH-2 and MONARCH-3	(35)
**Grade 3/4 Diarrhea (Letrozole)**	0.0012	MONALEESA-2	(5)
**Grade 3 Hepatotoxicity (Palbociclib)**	0	PALOMA-2	(3)
**Grade 3 Hepatotoxicity (Ribociclib)**	0.0051	MONALEESA-2	(5)
**Grade 3 Hepatotoxicity (Abemaciclib)**	0.0034	Safety report based on MONARCH-2 and MONARCH-3	(35)
**Grade 3 Hepatotoxicity (Letrozole)**	0.0008	MONALEESA-2	(5)
**Grade 4 Hepatotoxicity (Palbociclib)**	0	PALOMA-2	(3)
**Grade 4 Hepatotoxicity (Ribociclib)**	0.0012	MONALEESA-2	(5)
**Grade 4 Hepatotoxicity (Abemaciclib)**	0.0034	Safety report based on MONARCH-2 and MONARCH-3	(35)
**Grade 4 Hepatotoxicity (Letrozole)**	0	MONALEESA-2	(5)
**QTC > 500 mm/sec (Palbociclib)**	0	PALOMA-2	(3)
**QTC > 500 mm/sec (Ribociclib)**	0.0002	MONALEESA-2	(5)
**QTC > 500 mm/sec (Abemaciclib)**	0	Safety report based on MONARCH-2 and MONARCH-3	(35)
**QTC > 500 mm/sec (Letrozole)**	0	MONALEESA-2	(5)
**500> QTC >480 (Palbociclib)**	0	PALOMA-2	(3)
**500> QTC >480 (Ribociclib)**	0.002	MONALEESA-2	(5)
**500> QTC >480 (Abemaciclib)**	0	Safety report based on MONARCH-2 and MONARCH-3	(35)
**500> QTC >480 (Letrozole)**	0	MONALEESA-2	(5)

#### 2.2.2 Effectiveness

The effectiveness parameter needed for this model was measured on the QALY units. QALYs were calculated by multiplying the life years gained from a treatment modality by the utility value of a patient on this while being on that treatment modality (36). Utilities are numbers from 0 to 1 representing health-related quality (HR-QoL) of life, where 0 is death, and 1 is the full health (36). The utility values of PFS health states were summarized from HR-QoL studies and guidelines based on PALOMA-2, MONALEESA-2, and MONARCH-3 for the palbociclib, ribociclib, and abemaciclib treatment arms respectively (26–28). In addition, the PFS for letrozole-only patients was summarized from a meta-analysis for utility values for breast cancer patients, including patients on hormonal therapies like letrozole (29). The disutility values related to each of the major grade side effects (hematological toxicities, diarrhea and GI-related toxicities, and hepatotoxicity) were also summarized from the landmark trials. After progressing and moving to the PD status, all patients were assumed to have the same HR-QoL, with a utility value of 0.505 (31). The utility values used for this model input are summarized in **Table 1**.

#### 2.2.3 Transition probabilities

Transition probabilities controlled the movement between the health states. Transition probabilities were monthly based, and we calculated from the PALOMA-2, MONALEESA-2, MONARCH-3, and PO-25 trials for the palbociclib, ribociclib, abemaciclib, and letrozole monotherapy treatment arms respectively. First, the reported relative risks or odds ratios were converted to cumulative probabilities. Second, the cumulative probabilities were converted to rates. Third, the rates were converted to fixed time probabilities based on 1-month time intervals. The detailed calculation methods were followed based on the article published by Gidwani R. & Rusell L. (37). Since the majority of trials report only the number of deaths, or the odds of deaths, the deaths due to progression or other causes were not very clear. Therefore, it is assumed that 13% of the deaths is attributed to incidence whereas the remaining 87% percent is attributed to disease progression (38). Therefore, the ratios of PFS to death and PD to death were estimated from the odds of deaths in the clinical trials at 13% and 87% respectively. The detailed transition probabilities and the other model inputs are summarized in **Table 1** below.

### 2.3 Sensitivity analysis

A univariate deterministic sensitivity analysis (DSA) was run for the most effective CDK4/6 inhibitor over the others to address the effect of any uncertainties related to the main model inputs on the base-case cost-effectiveness conclusion. Costs were varied by 15% of the base-case cost. Whereas, utility values and transition probabilities were varied by 10% of the base-case cost, or if they were reported in terms of confidence intervals, the upper and the lower limits of the confidence intervals were used. For the transition probabilities and utilities, only variables with at least a 1% probability of base-case values were included in the sensitivity analysis. A tornado analysis was also conducted to investigate the variables associated with the most impact on the cost-effectiveness conclusion. The results of the tornado analysis were further summarized using a tornado diagram.

## 3 Results

When looking at the 10-year Markov’s model generated, it estimated an overall cost of letrozole 100,855 QAR with a generated 2.093 QALYs (25.12 quality-adjusted life months). As for Palbociclib, its overall estimated cost was 938,439 QAR, with a generated overall QALYs of 4.406 (52.87 quality-adjusted life months). Similarly, the overall cost for the ribociclib treatment arm was 879,873 QAR, and the overall QALYs was 4.4242. Lastly, for the abemaciclib treatment arm, the overall lifetime cost was 646,941 QAR for 4.2225. The three CDK4/6 inhibitors were first compared in their cost-effectiveness to letrozole monotherapy, and then to each other. Overall, the combination therapy with CDK4/6 inhibitors plus letrozole was shown to be cost-effective compared to letrozole alone at a WTP threshold of 448,785 QAR. That is, the ICER for palbociclib compared to letrozole monotherapy was 362,120 QAR/QALY with a significantly improved QALYs of 2.313 QALYs in total overall survival of which 0.922 in the PFS. For ribociclib combination therapy compared to letrozole monotherapy, the ICER was 334,170 QAR/QALY with an added 2.331 QALYs in the total overall survival, of which 1.097 is in the PFS state. Whereas, the ICER for abemaciclib plus letrozole versus letrozole monotherapy was 256,438 QAR/QALY with an increased overall QALYs of 2.13 QALY, of which 0.68 were in PFS.

In the cost-effectiveness scenario, the three CDK4/6 inhibitors were also compared to each other regarding their ICERs. At the current treatment costs, ribociclib was found to be dominant over palbociclib in terms of the overall cost and the lifetime QALYs. In addition, abemaciclib was found to be a cost-saving option compared to ribociclib with a reduced 0.202 QALYs but also a reduced cost of 232,932 QAR. In other words, ribociclib was a more clinically effective option but it needed of 1,154,843 QAR for each QALY gained. Similarly, abemaciclib plus letrozole was cost-saving compared to palbociclib plus letrozole with a reduced 0.1835 QALYs but also a 291,498 QAR saving. That implied an ICER of 1,588,545 QAR for each QALY gained when using palbociclib plus letrozole over abemaciclib plus letrozole. Therefore, although ribociclib was the most clinically effective option according to the model and it dominated palbociclib, abemaciclib was still considered the most cost-saving option according to the current threshold. The costs, effectiveness values, and ICERs of the three CDK4/6 inhibitors versus each other and versus letrozole monotherapy are illustrated in below **Table 2**.

**Table 2:** Base-case results for Markov’s Model Comparison for CDK4/6 Inhibitors vs Letrozole Monotherapy and vs Each Other.

Treatment Arm	Letrozole	Palbociclib	Ribociclib	Abemaciclib
Cost (QAR)				
**Total Cost**	100,855	938,439	879,873	646,941
**PFS Cost**	54,241	775,171	729,757	478,210
**PD Cost**	46,614	163,278	150,115	168,730
Effectiveness (QALY)				
**Total QALYs**	2.093	4.406	4.4242	4.2225
**QALYs in PFS**	1.538	2.46	2.635	2.212
**QALYs in PD**	0.555	1.946	1.789	2.010
ICER (QAR/QALY)				
**Vs. Letrozole Monotherapy**	–	362,120	334,170	256,438
**Vs. Palbociclib**	–	–	Ribo dominated	–
**Vs. Ribociclib**	–	Ribo. dominated	–	–
**Vs. Abemaciclib**	–	1,588,545	1,154,843	–

**Figure 1:**
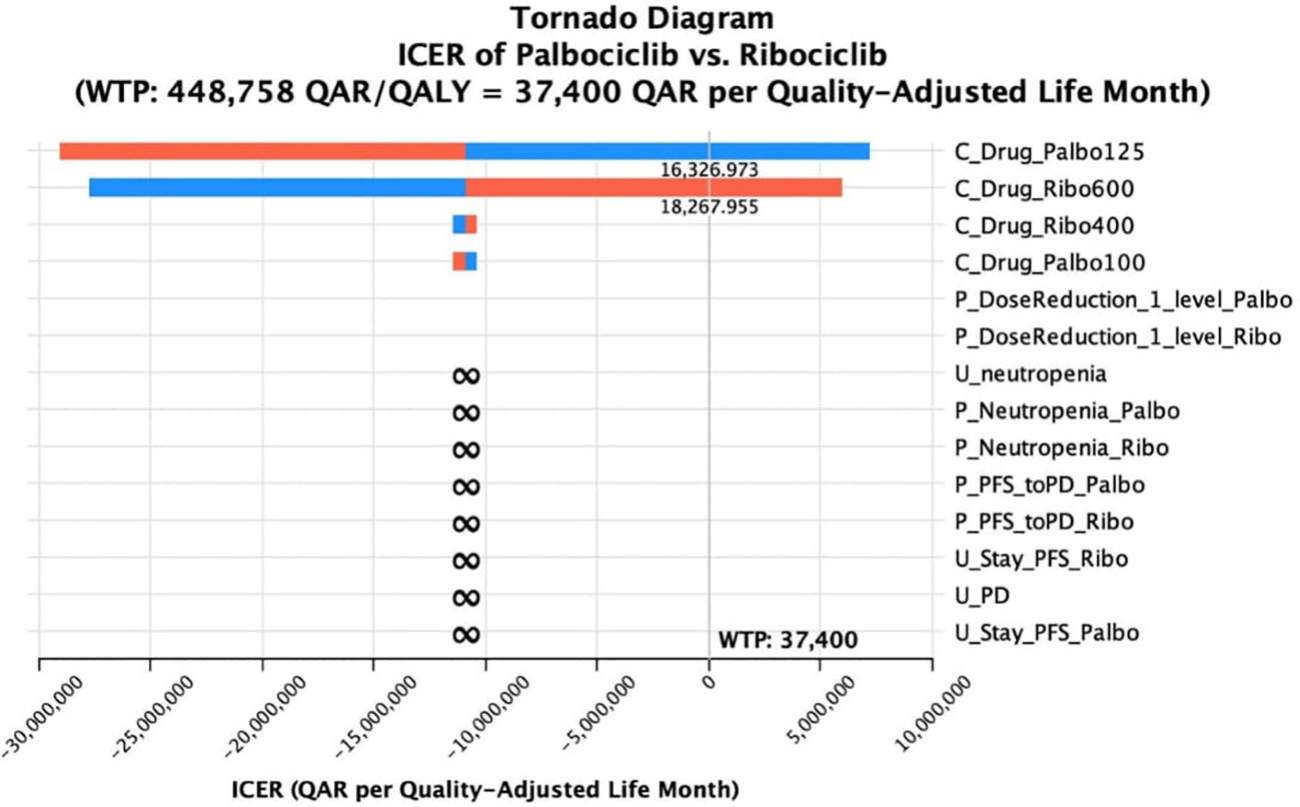
Tornado diagram of the incremental cost-effectiveness ratio (ICER) of palbociclib versus ribociclib at different ranges of uncertainties for selective variables.

**Figure 2:**
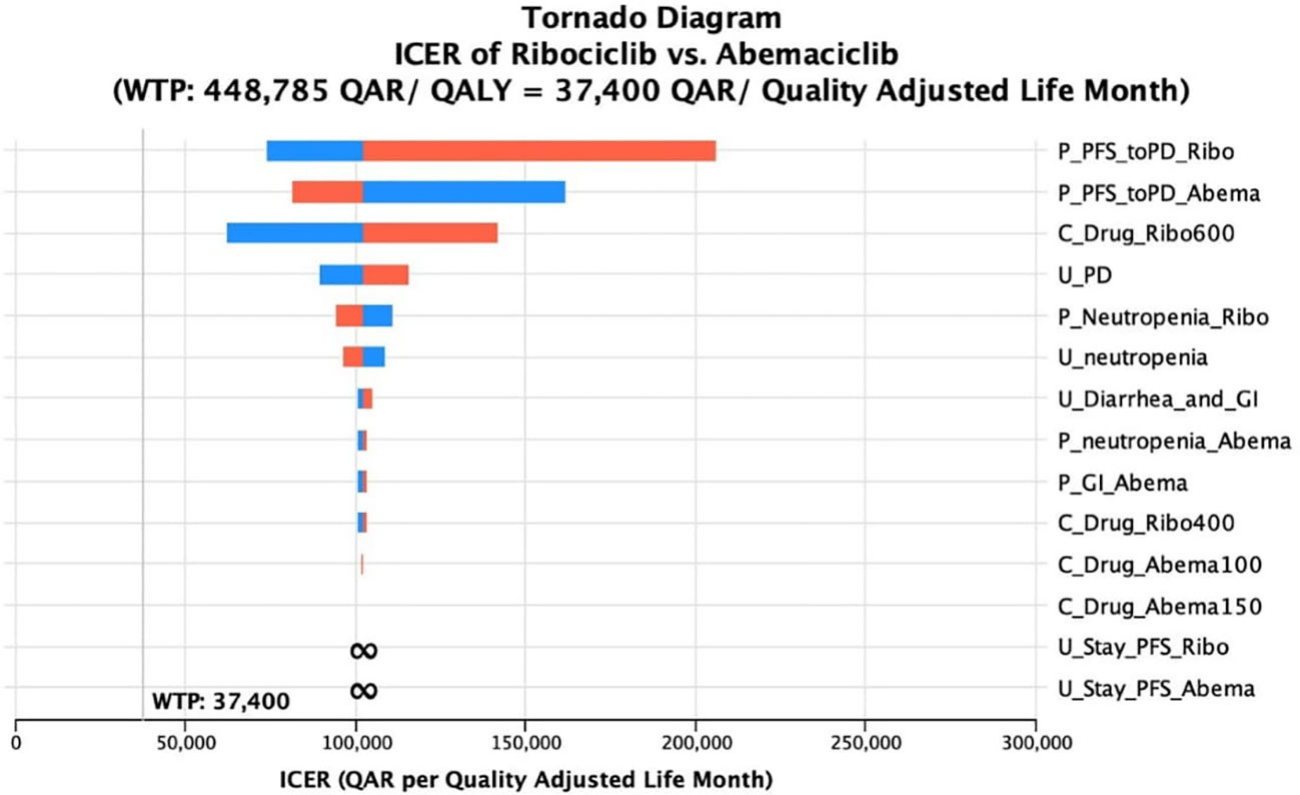
Tornado diagram of the incremental cost-effectiveness ratio (ICER) of Ribociclib versus Abemaciclib at different ranges of uncertainties for selective variables.

Lastly, the sensitivity analysis revealed that there were minor changes in the base-case conclusion with some factors only. That is, in the base-case Markov’s model conclusion, ribociclib dominated palbociclib in terms of costs and QALYs. While this conclusion remained consistent with the majority of the DSA inputs, raising the cost of ribociclib up to +15% was shown to slightly affect the results by having ribociclib as a non-cost-effective option compared palbociclib, with a cut-off of a ribociclib monthly cost raise starting from 18,267 QAR (+8.3% price increase); ICER started from 1,386,538 QAR/QALY added by the use of palbociclib. The same conclusion was obtained when decreasing the monthly cost of palbociclib by 9.1% or more; ribociclib was not cost-effective (ICERs started from 1,916,833 QAR/QALY). On the other hand, when decreasing the probability of moving from PFS to PD at the palbociclib treatment arm up to 10%, palbociclib was not dominated by ribociclib, but not cost-effective (ICERs started from 1,328,285 QAR/ QALY). The same conclusion was obtained when assuming an increase of the utility value for PFS health state in the palbociclib group to be equal to or more than 0.7515; ICER ≥2,686,512 QAR/QALY. Regarding the base-case conclusion of having abemaciclib as a cost-saving option compared to palbociclib, this conclusion remained robust against all the uncertainties associated with the different variables indicated in the sensitivity analysis. Lastly, regarding the base-case conclusion of having abemaciclib as a cost-saving option compared to ribociclib, there was no change in the base-case results in across all the uncertainty ranges, suggesting that the conclusion of having ribociclib not cost-effective to abemaciclib is robust. The DSA output and conclusions for ribociclib versus palbociclib are detailed in **Table 3**, and in **Table 4** for ribociclib versus abemaciclib. In addition, the effect of the DSA variables on the cost-effectiveness conclusions between palbociclib versus ribociclib and between ribociclib versus abemaciclib was further ranked using Tornado diagrams as illustrated in **Figures 1**, **2**.

**Table 3:** DSA outputs for palbociclib and ribociclib groups at each of the uncertainty parameters with the overall cost-effectiveness conclusions.

Uncertainty parameter	Uncertainty Range	Palbociclib		Ribociclib		Cost-Effectiveness Conclusion (robust/ sensitive)	Data Source for the Uncertainty Range
		Cost (QAR)	QALYs	Cost (QAR)	QALYs		
**Base-case**	0	938,439	4.406	879,873	4.4242	Ribociclib dominated Palbociclib	–
Uncertainties Associated with Costs							
**Palbociclib 125 mg cycle drug acquisition cost (full dose)**	- 15%	872,370	4.406	879,873	4.4242	Sensitive (Ribociclib is not cost-effective)	± 15% of base-case value
	+ 15%	1,049,509	4.406	879,873	4.4242	Robust	± 15% of base-case value
**Palbociclib 100 mg cycle drug acquisition cost (dose reduction by 1 level)**	- 15%	922,356	4.406	879,873	4.4242	Robust	± 15% of base-case value
	+ 15%	954,531	4.406	879,873	4.4242	Robust	± 15% of base-case value
**Ribociclib 600 mg cycle drug acquisition cost (full dose)**	- 15%	938,439	4.406	775,121	4.4242	Robust	± 15% of base-case value
	+ 15%	938,439	4.406	984,526	4.4242	Sensitive (Ribociclib is not cost-effective)	± 15% of base-case value
**Ribociclib 400 mg cycle drug acquisition cost (dose reduction by 1 level)**	- 15%	938,439	4.446	856,062	4.4242	Robust	± 15% of base-case value
	+ 15%	938,439	4.406	903,684	4.4242	Robust	± 15% of base-case value
Uncertainties Associated with Utility Values							
**Utility of staying at PFS (Palbociclib)**	0.7387	938,439	4.371	879,873	4.4242	Robust	Confidence interval boundaries (26)
	0.7627	938,439	4.446	879,873	4.4242	Robust till U≥0.7515 (At U<0.7515 palbociclib not dominated but still not cost-effective)	
**Utility of staying at PFS (Ribociclib)**	-10%	938,439	4.406	879,873	4.277	Robust till U≥0.714 (At U <0.714 (-7.75%) albociclib not dominated but still not cost-effective)	± 10% of base-case value
	+10%	938,439	4.406	879,873	4.671	Robust	
**Utility of PD**	0.45	938,439	4.314	879,873	4.348	Robust	Confidence interval boundaries (31)
	0.55	938,439	4.496	879,873	4.500	Robust	
**Utility of having grade 3/4 Neutropenia and blood relteted SEs**	-10%	938,439	4.3367	879,873	4.3415	Robust	± 10% of base-case value
	+10%	938,439	4.475	879,873	4.506	Robust	
Uncertainties Associated with Monthly Transition Probabilities							
**Moving from PFS to PD (Palbociclib)**	-10%	989,855	4.507	879,873	4.4242	Robust (palbociclib not cost-effective, but not dominated)	± 10% of base-case value
	+10%	892,859	4.316	879,873	4.4242	Robust	
**Moving from PFS to PD (Ribociclib)**	-10%	938,439	4.406	925,448	4.406	Robust	± 10% of base-case value
	+10%	938,439	4.406	839,121	4.319	Robust till P <0.021 (+7.4%) (At P≥0.021, palbociclib not cost-effective, but not dominated)	
**Dose reduction by 1 level (Palbociclib)**	+10%	932,255	4.406	879,873	4.4242	Robust	± 10% of base-case value
	-10%	944,623	4.406	879,873	4.4242	Robust	
**Dose reduction by 1 level (Ribociclib)**	+10%	938,439	4.406	898,655	4.4242	Robust	± 10% of base-case value
	-10%	938,439	4.406	861,091	4.4242	Robust	
**Grade 3/4 Neutropenia (Palbociclib)**	-10%	938,638	4.417	879,873	4.4242	Robust	± 10% of base-case value
	+10%	938,242	4.395	879,873	4.4242	Robust	
**Grade 3/4 Neutropenia (Ribociclib)**	-10%	938,439	4.406	881,605	4.438	Robust	± 10% of base-case value
	+10%	938,439	4.406	878,142	4.41	Robust	

**Table 4:** DSA outputs for Ribociclib Vs Abemaciclib groups at each of the uncertainty parameters with the overall cost-effectiveness conclusions.

Uncertainty parameter	Uncertainty Range	Abemaciclib		Ribociclib		Cost-Effectiveness Conclusion (robust/ sensitive)	Data Source for the Uncertainty Range
		Cost (QAR)	QALYs	Cost (QAR)	QALYs		
**Base-case**	0	646,941	4.2225	879,873	4.4242	Ribociclib was not cost-effective compared to abemaciclib	
Uncertainties Associated with Costs							
**Abemaciclib 150 mg BID cycle’s drug acquisition cost (full dose)**	- 15%	577,277	4.2225	879,873	4.4242	Robust	± 15% of base-case value
	+ 15%	716,604	4.2225	879,873	4.4242	Robust	
**Abemaciclib 100 mg BID cycle’s drug acquisition cost (dose reduction by 1 level)**	- 15%	577,277	4.2225	879,873	4.4242	Robust	± 15% of base-case value
	+ 15%	716,604	4.2225	879,873	4.4242	Robust	
**Ribociclib 600 mg cycle drug acquisition cost (full dose)**	- 15%	646,941	4.2225	775,121	4.4242	Robust	± 15% of base-case value
	+ 15%	646,941	4.2225	984,526	4.4242	Robust	
**Ribociclib 400 mg cycle drug acquisition cost (dose reduction by 1 level)**	- 15%	646,941	4.2225	856,062	4.4242	Robust	± 15% of base-case value
	+ 15%	646,941	4.2225	903,684	4.4242	Robust	
Uncertainties Associated with Utility Values							
**Utility of staying at PFS (Abemaciclib)**	-10	646,941	4.073	879,873	4.4242	Robust	± 10% of base-case value
	+10%	646,941	4.375	879,873	4.4242	Robust	
**Utility of staying at PFS (Ribociclib)**	-10%	646,941	4.2225	879,873	4.277	Robust	± 10% of base-case value
	+10%	646,941	4.2225	879,873	4.671	Robust	
**Utility of PD**	0.45	646,941	4.0125	879,873	4.348	Robust	CI boundaries (31)
	0.55	646,941	4.442	879,873	4.500	Robust	
**Utility of having grade 3/4 Neutropenia and blood related SEs**	-10%	646,941	4.195	879,873	4.3415	Robust	± 10% of base-case value
	+10%	646,941	4.250	879,873	4.506	Robust	
**Utility of having grade 3/4 diarrhea and other GI side effects**	-10%	646,941	4.129	879,873	4.396	Robust	CI boundaries (31)
	+10%	646,941	4.316	879,873	4.452	Robust	
Uncertainties Associated with Monthly Transition Probabilities							
**Moving from PFS to PD (Abemaciclib)**	-10%	675,380	4.302	879,873	4.4242	Robust	± 10% of base-case value
	+10%	621,762	4.152	879,873	4.4242	Robust	
**Moving from PFS to PD (Ribociclib)**	-10%	646,941	4.2225	925,448	4.406	Robust	± 10% of base-case value
	+10%	646,941	4.2225	839,121	4.319	Robust	
**Dose reduction by 1 level (Abemaciclib)**	+10%	646,941	4.2225	879,873	4.4242	Robust	± 10% of base-case value
	-10%	646,941	4.2225	879,873	4.4242	Robust	
**Dose reduction by 1 level (Ribociclib)**	+10%	646,941	4.2225	898,655	4.4242	Robust	± 10% of base-case value
	-10%	646,941	4.2225	861,091	4.4242	Robust	
**Grade 3/4 Neutropenia (Abemaciclib)**	-10%	646,941	4.225	879,873	4.4242	Robust	± 10% of base-case value
	+10%	646,941	4.219	879,873	4.4242	Robust	
**Grade 3/4 Neutropenia (Ribociclib)**	-10%	646,941	4.2225	881,605	4.438	Robust	± 10% of base-case value
	+10%	646,941	4.2225	878,142	4.41	Robust	

## 4 Discussion

The present study aimed to evaluate the cost-effectiveness of CDK4/6 inhibitors in combination with letrozole as a first-line treatment for hormonal receptor-positive and HER-2-negative advanced breast cancer in addition to evaluating their cost-effectiveness versus one another. Our analysis utilized a Markov model to simulate the long-term economic and clinical outcomes of this treatment strategy. From a cost-effectiveness perspective, the results suggest that the addition of CDK4/6 inhibitors to letrozole is associated with increased costs, primarily due to the high acquisition costs of these targeted therapies. However, the incremental cost-effectiveness ratio (ICER) falls within the commonly accepted WTP, indicating that the combination therapy with any of the three FDA-approved CDK4/6 inhibitors is a cost-effective option compared to letrozole alone. In addition, for the cost-effectiveness comparison between the three CDK4/6 inhibitors, ribociclib generated the highest QALYs; however, it was not cost-effective compared to abemaciciclib, but it was dominant over palbociclib. Of note, the literature about WTP threshold in oncology interventions is greatly expanding (39). The variations can be seen with respect to not only the types of interventions and diseases under study, but also the settings and methods of health-economic evaluations being used (39). In this evaluation, we compared our ICERs to a WTP of 1.5 GDP per capita, which is unlike the normally adopted 3 GDP per capita recommended by the WHO. This was adapted based on clinical and economic experts’ opinions due to the high GDP per capita of Qatar which can reach to more than 3 times than many other countries (40). Therefore, the results are more reasonable and tailored to the healthcare payer perspective based on the economic setting.

First, regarding the cost-effectiveness of CDK4/6 inhibitors versus letrozole monotherapy, this conclusion may diverge from some previous research (16). We reason it is mainly due to the variation of the model assumptions and parameterization. That is, our Markov model was constructed based on the best available up-to-date evidence from clinical trials, but variations in model assumptions and parameterization can lead to divergent results. Differences in the selection of transition probabilities, time horizons, utility values, and WTPs may contribute to disparities between our study and prior investigations. For example, in one study that compared the cost-effectiveness of palbociclib plus letrozole to letrozole monotherapy and found palbociclib was not cost-effective, they used the data from PALOMA-1 (41)and PALOMA-2 (3) trials which are less updated (42). In our analysis for palbociclib plus letrozole versus letrozole monotherapy, we used only the PALOMA-2 trial which has the mature PFS data compared to PALOMA-1. Similarly, for one of the studies that compared ribociclib combination therapy to letrozole monotherapy, we compared the ICERs to our WTP threshold of 1.5 GDP (448,785 QAR), but for that study, the maximum WTP threshold was $53,384 (195,385 QAR) which is 43% of our threshold, so it was not cost-effective at this threshold (43). Similar Markov-based inputs and different WTPs were used in the studies that differed from us for that outcome. However, other factors such as different Model perspectives and different population characteristics could be potential reasons too.

The second objective of our analysis was to compare the cost-effectiveness of the three CDK4/6 inhibitors to each other. Ribociclib dominated palbociclib in terms of costs and effectiveness. This finding was consistent with the previous research done earlier by our research group based on real-world data (RWD) retrieved from patients on palbocilcib and ribociclib in Qatar (18). In addition, it was also consistent with the findings of other cost-effectiveness evaluations done in other parts of the world such as Spain, the USA, and the UK (13, 17, 19). Nonetheless, ribociclib failed to be a cost-effective option compared to abemaciclib. This may be due to the huge difference in drug acquisition cost between the two drugs where abemaciclib’s drug acquisition cost is almost 1/2 of the ribociclib’s which impacts the overall monthly cost of both. While yielding a very similar number of gained QALYs (4.222 for abemaciclib versus 4.424 for ribociclib), that difference in QALYs gained was not worth the added cost from the cost-effectiveness perspective. The finding of our study that abemaciclib is a cost-saving option over ribociclib was not consistent with other two published findings from the two studies that we could identify to address the cost-effectiveness of abemaciclib plus aromatase inhibitors versus ribociclib plus aromatase inhibitors in HR+/HER2- advanced breast cancer patients (44, 45). That is, the first study that was conducted in Brazil found that ribociclib was the most cost-effective medication followed by abemaciclib followed by palbociclib. Although that study has used a similar model input source, we noticed that their ribociclib cost was the lowest followed by abemaciclib followed by ribociclib, with ribociclib 20% less than abemaciclib (44). Therefore, we reason that the difference in conclusion to the different drug costs between our settings and theirs. The second study was conducted in the USA; however, it was published only in an abstract form which prevented us from digging deeper to investigate the reasons for the difference in conclusions (45). Lastly, our study showed that palbociclib was not also a cost-effective option compared to abemaciclib. This was consistent to much extent with the findings of the studies that we could identify by either having abemaciclib as a more cost-effective option than palbociclib or a dominant option (44, 46).

Lastly, since ribociclib was the most effective option, we underwent a deterministic sensitivity analysis for ribociclib versus palbociclib, and for ribociclib versus abemaciclib. Our results remained robust against all the uncertainty range for ribociclib versus palbociclib except for the reduction of the drug acquisition cost component during the palbociclib 125 mg PFS health status, and the increase of the price of the drug acquisition during the ribociclib 600 mg PFS health status. This suggested that for palbociclib to be a cost-saving option compared to ribociclib, the drug acquisition cost needs to drop by at least 9%. Regarding the conclusion of ribociclib to abemaciclib, this conclusion has not been changed by all uncertainties. We reason this mainly again to the great difference in the drug acquisition cost which is approximately 50% while the low number of QALY difference (0.2 QALYs).

Several notable strengths characterize our current study. First of all, to our knowledge, it is the first comparative comprehensive pharmacoeconomic evaluation for the three CDK4/6 inhibitors and letrozole monotherapy in the first-line use of HR+/HER2- advanced breast cancer. Second, we employed a comprehensive Markov model to simulate the long-term economic and clinical outcomes of the treatment strategies under consideration with transparent assumptions and model inputs for reproduction. In addition, we conducted extensive sensitivity analyses, varying key parameters within clinically relevant ranges. The consistency of our results under different scenarios enhances the reliability of our conclusions and underscores the stability of the observed cost-effectiveness outcomes. However, it is crucial to acknowledge and address certain limitations that may impact the interpretation and generalizability of our findings. First, while using data from clinical trials enhances generalizability, the study's reliance on clinical trial data for certain clinical inputs necessitates an extrapolation of short-term trial results to long-term outcomes which introduces inherent uncertainties, as the long-term efficacy and safety profiles of treatments may differ from the observed trial durations. However, we could address this potential limitation by conducting a deterministic sensitivity analysis for the variables of uncertainty. One more limitation is related to the Model’s generalizability. This is because the model was based on costs retrieved from a Qatari hospital and analyzed to a WTP from a Qatari perspective. It is noteworthy to mention that while generalizability is a general limitation for all health economics analyses, Qatar economic system differs from many countries in the world in terms of the GDP. While the current average worldwide GDP is about 12,703 USD per capita, the GDP in Qatar reaches to USD 87,661 per capita (40). As discussed earlier, this has affected the WTP to which the cost-effectiveness was evaluated. Therefore, only countries with relatively similar GDP per capita can generalize the current findings of the study to their content.

While this study provided comprehensive cost-effectiveness evidence about CDK4/6 inhibitors in the first-line use in HR+/HER2- advanced breast cancer, future research should go beyond traditional cost-effectiveness studies and delve into affordability, budget impact, market dynamics, and comparative effectiveness to provide a comprehensive understanding of the role and potential market uptake of CDK4/6 inhibitors in the treatment of advanced breast cancer. These efforts will not only inform clinical decision-making but also facilitate the development of sustainable and equitable healthcare strategies. In addition, future comprehensive health economic evaluation of CDK4/6 inhibitors versus other similarly expensive medications in different clinical settings such as chemotherapy plus bevacizumab in the second-line treatment in case of imminent organ failure or in comparison with inhibitors of the enzyme poly ADP ribose polymerase (PARP) in cases of the presence of HR+/HER2- BRCA1 and/or BRCA2 mutations should be studied in more detail. Lastly, in order to have a holistic picture of the cost-effectiveness of CDK4/6 inhibitors worldwide, there should be more cost-effectiveness and cost-benefit evaluations for CDK4/6 inhibitors from developing countries and countries with emerging economies too. The available evaluations, such as the current one, are from countries with developed economies, and accordingly, is not generalizable to countries with developing economies.

## 5 Conclusions

Our evaluation of the cost-effectiveness of CDK4/6 inhibitors in combination with letrozole as a first-line treatment for HR+/HER2- advanced breast cancer provided valuable insights into the economic and clinical considerations of these therapeutic strategies. Ribociclib was the most effective option among the CDK4/6 inhibitors considered in this study. It not only demonstrated superior clinical outcomes but also dominated palbociclib in terms of cost-effectiveness. However, ribociclib fell short of being cost-effective compared to abemaciclib, where abemaciclib was a cost-saving treatment compared to ribociclib. This study can guide clinicians and decision-makers regarding the best cost-effective use of CDK4/6 inhibitors in the first-line use of HR+/HER2- advanced breast cancer. Nonetheless, due to the limitation of generalizability specially for countries with developing economies or different healthcare systems than Qatar, similar evaluations should be carried out in different settings to assure a holistic guidance for the most cost-effective use of CDK4/6 inhibitors in HR+/HER2- advanced breast cancer. In addition, further research into the economic impact of treatment sequencing and identifying patient subgroups that derive maximal benefit from specific CDK4/6 inhibitors would be needed to help in personalizing treatment strategies.

## Data availability statement

The data analyzed in this study is subject to the following licenses/restrictions: The data presented in this study are not public as they are extracted from the hospital's electronic health records. They can be provided anonymously by request from the corresponding author. Requests to access these datasets should be directed to AH, ahamad6@hamad.qa.

## Ethics statement

The studies involving humans were approved by the Institutional Review Board, Hamad Medical Corporation (approval no. MRC-02-22-333). The studies were conducted in accordance with the local legislation and institutional requirements. Written informed consent for participation was not required from the participants or the participants' legal guardians/next of kin in accordance with the national legislation and institutional requirements.

## Author contributions

AH: Conceptualization, Formal analysis, Funding acquisition, Investigation, Methodology, Project administration, Resources, Supervision, Validation, Visualization, Writing – original draft, Writing – review & editing. SE: Conceptualization, Data curation, Formal analysis, Investigation, Methodology, Supervision, Visualization, Writing – original draft, Writing – review & editing. NA-Z: Conceptualization, Data curation, Formal analysis, Investigation, Methodology, Software, Validation, Visualization, Writing – original draft. MM: Conceptualization, Formal analysis, Supervision, Visualization, Writing – review & editing, Methodology. SB: Conceptualization, Methodology, Supervision, Validation, Writing – review & editing.
